# A 41-Year-Old Patient with a Rare Cause of Severe Abdominal Sepsis Misdiagnosed as PID

**DOI:** 10.1155/2018/9561798

**Published:** 2018-04-10

**Authors:** Anastasia Mikuscheva, David Becker, Mark Thompson-Fawcett

**Affiliations:** ^1^Department of Gynecology and Obstetrics, Dunedin University Hospital, Dunedin, New Zealand; ^2^Department of Surgery, Dunedin University Hospital, Dunedin, New Zealand

## Abstract

Infectious pelvic inflammatory disease is a common condition and a frequent cause of abdominal pain in a young female patient. In a patient who has not completed family planning, the diagnosis is often made with a low threshold and treatment started on a low suspicion of diagnosis to avoid a negative impact on fertility. Here, we present a case of a 41-year-old woman who was misdiagnosed with infectious pelvic inflammatory disease and treated ineffectively with antibiotics when the underlying condition of her persistent abdominal pain was a midgut neuroendocrine tumor that had caused bowel perforation and formation of an abscess in the pouch of Douglas.

## 1. Introduction

Pelvic inflammatory disease (PID) is defined as an infection-induced inflammation of the female upper reproductive tract. The hallmark of the diagnosis is pelvic tenderness combined with inflammation of the lower genital tract. Acute (≤30 days' duration), clinically diagnosed PID is caused by spontaneous ascension of microbes from the cervix or vagina to the endometrium, fallopian tubes, and adjacent structures. More than 85% of infections are due to sexually transmitted cervical pathogens (*C. trachomatis* and *N. gonorrhoeae*) or bacterial vaginosis-associated microbes, and approximately 15% are due to respiratory or enteric organisms that have colonized the lower genital tract. The symptoms associated with acute PID include pelvic or lower abdominal pain of varying severity, abnormal vaginal discharge, intermenstrual or postcoital bleeding, dyspareunia, and dysuria. Systemic manifestations are not a prominent feature of PID. Occasionally, right-upper-quadrant pain suggestive of inflammation and adhesion formation in the liver capsule (perihepatitis or the Fitz-Hugh–Curtis syndrome) can accompany PID[1]. 

Neuroendocrine tumors (NETs) are slow-growing epithelial tumors with predominant neuroendocrine differentiation. Being a rare form of neoplasm, they are frequently not considered in the differential diagnosis. Clinical manifestations are often nonspecific and may include abdominal pain, bowel obstruction, diarrhoea, weight loss, and gastrointestinal bleeding [2].

Here, we present a case of a female patient with nonspecific abdominal symptoms, raised inflammatory markers, and inconclusive imaging initially misdiagnosed with PID. The correct diagnosis was obtained histologically after a second acute presentation with abdominal symptoms that led to a diagnostic laparoscopy and eventually laparotomy and tumor resection.

## 2. Case Report

Miss T is a 41-year-old Caucasian woman who was admitted to the intensive care unit of our hospital for an episode of severe diabetic ketoacidosis. The day prior to admission, the patient had been unwell and vomiting and was eventually found unconscious and admitted to hospital. The patient's medical history was significant for poorly controlled type 1 diabetes on insulin, diabetic retinopathy, depression, anxiety, and inactive hepatitis C. She was a previous intravenous drug user, was on buprenorphine treatment with intermittent abuse of benzodiazepines and opioids, and was a cigarette smoker. Her surgical history included laparoscopic bilateral tubal ligation and a LLETZ conisation for a CIN 2 cervical lesion.

**Figure 1:**
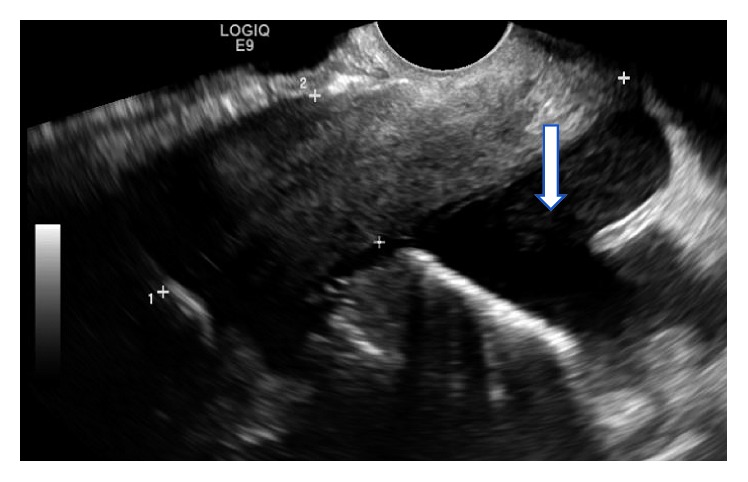
Complex free fluid.

On admission, her temperature was 29.4°C, her respiratory rate was 28, pulse was 75, BP was 134/87 mmHg, and GCS was 10. Laboratory testing showed severe metabolic acidosis and acute renal failure and inflammation. The patient was started on treatment as per hospital DKA protocol and meropenem intravenously, given her severely raised inflammatory markers with an unclear infection focus. After stabilisation of her status, the patient was discharged to the medical ward. She became febrile several days later and began reporting lower abdominal pain, nausea, and intermenstrual vaginal bleeding. Rising inflammatory markers were noted. She was reviewed by the surgical team, found to be constipated, and treated with laxatives. A pelvic ultrasound was organised to locate the infection focus. This reported “complex fluid within the pouch of Douglas” (Figure 1), with pelvic infection representing the most likely cause. The patient was reviewed by the gynaecology team and switched to oral co-amoxicillin.

On this treatment, the inflammatory markers, that had begun to drop on meropenem, started to rise again. The antibiotic regime was hence changed to oral ciprofloxacin and metronidazole. During her hospital admission, the patient complained repeatedly of nausea, which was considered a side effect of metronidazole. The patient was discharged home on oral antibiotics when she was afebrile and feeling better.

**Figure 2:**
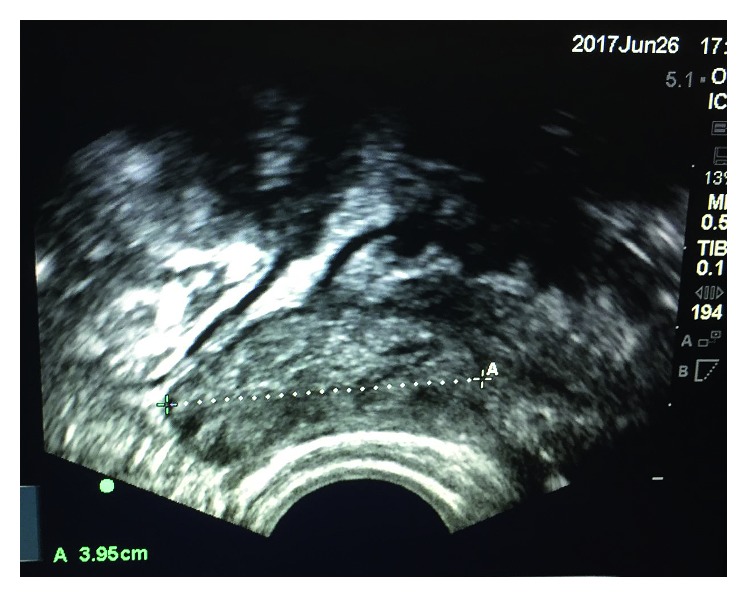
Mass in the pouch of Douglas.

**Figure 3:**
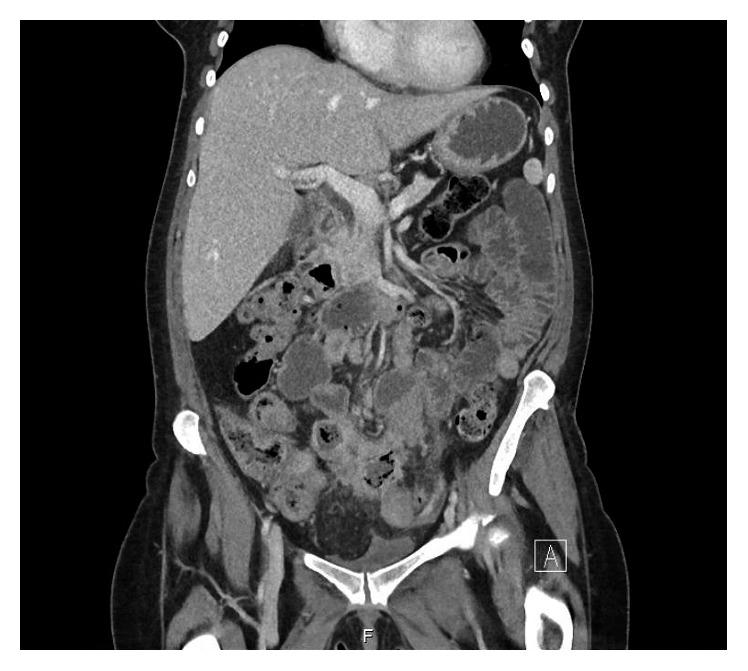
CT scan with unspecific inflammatory changes.

Three weeks later, the patient returned to the emergency department of our hospital reporting predominantly right iliac fossa pain, persistent nausea, and anorexia. On a transvaginal bedside ultrasound scan, a hyperechogenic mass was seen in the pouch of Douglas which, given the previous diagnosis of PID, was interpreted as an adnexal mass (Figure 2). The scan had to be abandoned due to patient discomfort. To further evaluate the mass, an abdominal CT scan was organised. The transvaginal pictures were reviewed with the radiological colleagues and however could not be correlated with the CT findings which reported the appearances in the pelvis as nonspecific. There were some pockets of fluid and some thickened loops of small bowel, which were potentially consistent with secondary involvement of small bowel in a pelvic inflammatory process (Figure 3). The patient was rediscussed with the surgical team; however, their advice was that a surgical cause for the patient's presentation was unlikely. A diagnostic laparoscopy was organised by the gynaecological team with the surgical team on standby. Whilst awaiting the procedure, the patient started vomiting profusely and required a nasogastric tube placement.

On diagnostic laparoscopy, normal internal genitalia were seen, however adherent bowel tissue became apparent; the surgical team was called, and the operation was converted to a midline laparotomy. There was a complex inflammatory mass containing an abscess cavity in the upper portion of the pelvis, and a 10 to 15 cm segment of the ileum was completely gangrenous and necrotic but still structurally intact. Surprisingly, there was no small bowel obstruction, and the small bowel contents appeared to be passing through the lumen of the gangrenous ileum. There was an enlarged node in the ileal mesentery associated with significant fibrosis raising the suspicion that there may be a NET nodal metastasis causing occlusion of the mesenteric vessels. The inflammatory mass was removed, an ileocolic resection was performed, and a double-barrelled stoma was created.

The patient recovered well from the operation and was discharged home a week later. The histology report showed the presence of a well-differentiated ileal neuroendocrine primary tumor that was not apparent on laparotomy. One of the 20 mesenteric lymph nodes present in the resected material was metastatic. On postoperative review of the CT scan, neither the primary tumor nor the nodal mass was distinguishable from the inflamed tissue.

The patient was discussed at a multidisciplinary tumor meeting, and a staging CT chest-abdomen-pelvis was performed. Fortunately, this showed no evidence of local recurrence or metastases. The patient currently remains well and has since undergone stoma reversal surgery.

## 3. Discussion

PID is a common cause for lower abdominal pain in women. Risk factors include age < 25 years, age at first sexual intercourse < 15 years, lower socioeconomic status, being single, a self-reported history of a sexually transmitted disease, and exposure to *C trachomatis* [3].

In our case, the patient was a previous drug user and had poor socioeconomic status with an inactive hepatitis C infection and a history of LLETZ conisation for CIN 2 which classified her as a high-risk patient for sexually transmitted disease [3]. Moreover, seeing as her abdominal pain was accompanied by fever, raised inflammatory markers, intermenstrual bleeding, and “complex”, free fluid in the pouch of Douglas, a diagnosis of PID was readily made. The fact that she was in a stable relationship and had bilateral tubal ligation was considered as risk-reducing factors; however, it was insufficient to refute the diagnosis. On reflection, the history and clinical course, combined with failure to resolve on standard antibiotic treatment, were not entirely typical for PID. In addition, the imaging was suggestive but not conclusive of PID. Earlier consideration of a wider differential diagnosis may have led to an earlier laparoscopy.

NETs are composed by neuroendocrine cells which are scattered through the mucosa of the gastrointestinal tract. These cells get their name from their ability to express some proteins classically attributable to neural cells, such as neuron-specific enolase and synaptophysin, and to their capacity to produce hormones, such as somatostatin, substance P, and vasoactive intestinal peptide. It is estimated that 64% of all NETs originate in the gastrointestinal tract and 28% originate in the lungs and bronchi. Within the gastrointestinal tract, the most frequently affected sites are the small intestine (29%), rectum (14%), stomach (5%), and appendix (5%) [4, 5].

NETs are a rare tumor entity, and 60% of patients with NETs are asymptomatic and discover their tumors incidentally during medical workup for something else [4], that is, irritable bowel syndrome [6]. At diagnosis, if they are not an incidental finding, lesions are commonly > 2 cm, with invasion of muscularis propria and metastases to regional lymph nodes. Midgut NETs are associated with mesenteric fibrosis, which can compress mesenteric vessels, and cause bowel ischemia and malabsorption, even in the absence of an obvious abdominal mass [7]. Multiple lesions may be found in up to 40% of cases [8]. The annual incidence of NETs has risen in the last years from 40 to 50 cases per million, probably not due to a real increase in incidence but rather due to better diagnostic tools that have become progressively available [9].

The evaluation of a young female patient in the emergency department often creates a diagnostic dilemma that has been highlighted repeatedly in the literature [10–17], to name but a few examples. However, in all those cases, CT or ultrasound imaging was helpful in obtaining the correct diagnosis. In our case, an ultrasound scan performed during the patient's first presentation showed what was described as “complex fluid” but was unable to visualize any masses. During her 2nd presentation, a transvaginal scan showed a mass in the pouch of Douglas which, given the previous diagnosis of PID, was interpreted as an adnexal mass (Figure 2). Retrospectively, it was recognized to be the abscess which was part of the inflammatory mass seen intraoperatively, not the tumor itself. In our hospital, bedside ultrasound pictures cannot be saved onto the electronic system and accessed by the radiology department. Printouts of the images were discussed with the radiology trainee who was present at the hospital but not the consultant who was not on site. Despite prior discussion, the transvaginal ultrasound images could not be correlated on the CT scan which showed nonspecific bowel changes only and thus did not contribute to obtaining the correct diagnosis. Our case demonstrates that despite the advances in the development of diagnostic tools, meticulous history taking and clinical examination remain indispensable and cannot be replaced by imaging only.

When the patient kept complaining of gastrointestinal symptoms and responded poorly to antibiotic treatment, alternative diagnoses were not sufficiently considered. Her persistent nausea, for instance, was wrongly attributed to metronidazole treatment, and she was discharged home with significantly raised inflammatory markers. Interestingly, she developed profuse vomiting only on the 3rd day of her 2nd presentation and was able to pass stool regularly until her operation. This is likely due to the fact that her bowel lumen remained patent at least intermittently for a long time despite the space occupying tumor, ischemia, and abscess formation. The bowel perforation which was evident during the operation finally interrupted the bowel passage. It is likely that this patient had at least partial ischemia during her first hospitalisation and then developed an infarction of a short segment of ileum causing the symptoms which led to her second presentation.

Our case was complicated by the patient's type 1 diabetes. DKA symptoms can mimic acute abdominal infection and per se cause abdominal pain and nausea. Misdiagnosis of DKA as PID has been reported [18]. It is not clear whether the patient's DKA was exacerbated by catecholamine production by the ileal tumor or whether her poorly controlled type I diabetes had led to DKA. It is possible that the DKA caused a transient episode of hypotension which contributed to bowel ischemia and necrosis or that the increased circulating levels of glucagon and catecholamines or other hormones accompanying ketoacidosis impeded gastrointestinal motility [19].

## 4. Conclusion

It is important to question your diagnosis, especially if a patient continues to exhibit persistent symptoms despite adequate therapy for the initial diagnosis. Common things are common; however, rarer differential diagnosis must be considered in the patient who remains unresponsive to therapy. Finally, in case of diagnostic uncertainty, exploratory surgery will often be important to resolve the problem and should be undertaken without undue delay.
